# Efficient Quantum Simulation of an Anti-*P*-Pseudo-Hermitian Two-Level System

**DOI:** 10.3390/e22080812

**Published:** 2020-07-24

**Authors:** Chao Zheng, Jin Tian, Daili Li, Jingwei Wen, Shijie Wei, Yansong Li

**Affiliations:** 1 Department of Physics, College of Science, North China University of Technology, Beijing 100144, China; tianjin191988@163.com (J.T.); 15680600858@163.com (D.L.); 2 State Key Laboratory of Low-Dimensional Quantum Physics and Department of Physics, Tsinghua University, Beijing 100084, China; wjw17@mails.tsinghua.edu.cn (J.W.); liangqq1989626@163.com (S.W.); ysli@tsinghua.edu.cn (Y.L.); 3 Beijing Academy of Quantum Information Sciences, Beijing 100193, China; 4 Frontiers Science Center of Quantum Information, Beijing 100084, China

**Keywords:** quantum simulation, duality quantum algorithm, non-Hermitian, anti-*P*-pseudo- Hermitian, anti-PT-symmetry

## Abstract

Besides Hermitian systems, quantum simulation has become a strong tool to investigate non-Hermitian systems, such as PT-symmetric, anti-PT-symmetric, and pseudo-Hermitian systems. In this work, we theoretically investigate quantum simulation of an anti-*P*-pseudo-Hermitian two-level system in different dimensional Hilbert spaces. In an arbitrary phase, we find that six dimensions are the minimum to construct the anti-*P*-pseudo-Hermitian two-level subsystem, and it has a higher success probability than using eight dimensions. We find that the dimensions can be reduced further to four or two when the system is in the anti-PT-symmetric or Hermitian phase, respectively. Both qubit-qudit hybrid and pure-qubit systems are able to realize the simulation, enabling experimental implementations in the near future.

## 1. Introduction

Enlightened by Feynman’s thinking that simulates physics using nature [1], plenty of scientists manage to turn it into reality. As near-term quantum computers are available in some ways [2,3], quantum simulation has opened an effective and efficient way to investigate novel systems and phenomena related to both Hermitian [4,5,6,7,8,9,10,11] and non-Hermitian (NH) [12,13,14,15,16,17,18,19,20,21,22] Hamiltonians. Especially for the later one, quantum simulation has become the main method for experimental investigations in quantum level, which a non-Hermitian system can be constructed, operated, and observed in a subspace of a controllable quantum system.

Typical non-Hermitian Hamiltonians include PT-symmetric *H*, anti-PT-symmetric *H* and pseudo-Hermitian *H*, attracting considerable attention by various motivations such as extension of fundamental quantum theory, exceptional points, novel optical properties, etc. Since Hermiticity guarantees the eigenvalues of the Hamiltonian of a quantum system real, it ensures quantum mechanics physically. However, it is a sufficient but not essential condition for the reality of observables. A class of Hamiltonians with PT-symmetry, which commute with the joint operation parity *P* and time-reversal operator *T*, was found to be able to keep the eigenvalues of *H* real in the exact PT phase [23]. Since then, PT quantum mechanics has been extensively investigated theoretically [24,25,26,27,28,29,30,31,32,33,34,35,36,37,38,39,40,41,42,43,44] and experimentally [12,13,14,45,46,47,48]. In fact, *P*-pseudo-Hermiticity was pointed out to be a sufficient and necessary condition to keep the spectrum of a non-Hermitian Hamiltonian purely real [49,50,51], and both the theory and applications are developed further [52,53,54,55,56,57]. Recently, a class of Hamiltonians with anti-PT-symmetry started to attract researchers’ attention for its novel properties and applications [58,59,60,61,62,63,64,65,66,67,68]. Hamiltonians with anti-PT symmetry are anti-commutative with the joint operation PT, showing appealing features, such as balanced positive and negative index [58], constant refraction [64], novel property of information flows [21], etc. Given that the development and relations of PT-symmetry, *P*-pseudo-Hermiticity and anti-PT-symmetry, it naturally extends to a Hamiltonian with anti-*P*-pseudo Hermiticity.

Several proposals have been given to simulate PT-symmetric systems [12,14,18,19,28,69], *P*-pseudo-Hermitian systems, [18,55,57], and anti-PT-symmetric systems [20,21] based on different theories. However, quantum simulation designed for anti-*P*-pseudo-Hermitian systems is absent. In this work, we investigate quantum simulation of a time-independent anti-*P*-pseudo-Hermitian two-level system both in an arbitrary phase and in special phases, respectively. Because of the non-Hermiticity, the system cannot be simulated by normal methods designed for Hermitian systems. Therefore, we construct the non-Hermitian subsystem in a larger Hilbert space using the method of duality quantum computing, and simulate the time evolution. Furthermore, the efficiency and successful probability to simulate a generalized anti-PT-symmetric system in the previous work [20,21] are improved by this work. We design quantum circuits for both qubit-qudit hybrid and pure-qubit quantum computers. Therefore, implementations can be expected in real quantum systems, and properties of the anti-*P*-pseudo-Hermitian system can be demonstrated experimentally by quantum simulation.

## 2. Anti-P-Pseudo-Hermitian Two-Level System

Similar to the relation between PT and anti-PT symmetry, an anti-*P*-pseudo-Hermitian (anti-PPH) Hamiltonian can be obtained by a *P*-pseudo-Hermitian Hamiltonian times the imaginary unit *i*, satisfying

(1) $$H^{†}=-PHP,$$

where *P* is the parity operator. For a time-independent two-level case, $P=\left(\begin{array}{cc} 0 & 1\\ 1 & 0 \end{array}\right)$ and the Hamiltonian has an explicit form of

(2) $$H=i\left(\begin{array}{cc} re^{i\theta } & s\\ u & re^{-i\theta } \end{array}\right),$$

where *r*, *s*, *u* and θ are four independent real dynamic parameters of the system. The eigenvalues of *H*, which are decided by the four dynamic parameters, equate to $\varepsilon_{\pm }=i\left(rcos\theta \pm \sqrt{us-r^{2}\mathrm{si}n^{2}\theta }\right)$. We set the difference of the two eigenvalues as

(3) $$\omega =2\sqrt{r^{2}\sin^{2}\theta -us}.$$

If ω is equal to zero, it is not difficult to check that only one independent eigenvector exists and it is called the exceptional point [70] of the *H* in the parametric space.

**Figure 1:**
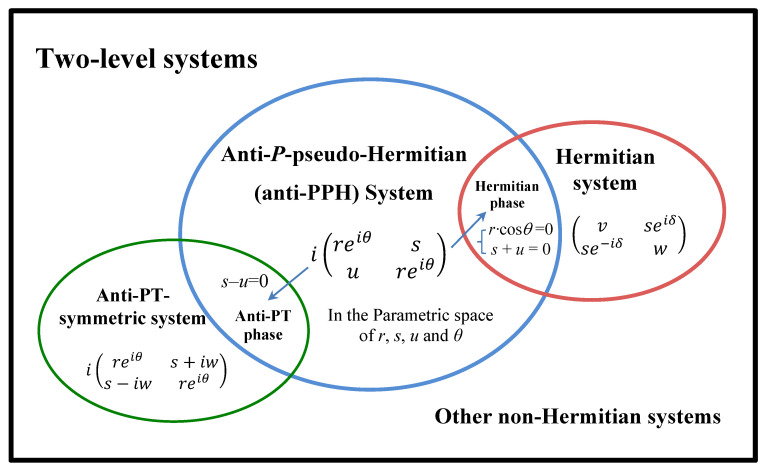
Sets of anti-*P*-pseudo-Hermitian, anti-PT-symmetric, and Hermitian two-level systems. The blue ellipse is the general set of anti-*P*-pseudo-Hermitian two-level Hamiltonians which are decided by four dynamic parameters *r*, *s*, *u* and θ. When the dynamic parameters are provided some limitations, the anti-PPH Hamiltonian enters into the anti-PT-symmetric or Hermitian phase. Therefore, there is an intersection between the blue ellipse and the green ellipse (anti-PT-symmetric set) or the red ellipse (Hermitian set).

Although *H* in Equation (3) is anti-*P*-pseudo Hermitian in general, it may has other symmetries in some special phases (see Figure 1). For example, when s−u=0 (the intersection of the blue and green ellipses in Figure 1), *H* becomes

(4) $$H^{APT}=i\left(\begin{array}{cc} re^{i\theta } & s\\ s & re^{-i\theta } \end{array}\right),$$

which also satisfies the relation that PTH+HPT=0, indicating that it has anti-PT symmetry in this phase. When rcosθ=0 and s+u=0 (the intersection of the blue and red ellipses in Figure 1), *H* becomes Hermitian.

Notice that *H* in Equation (2) is not Hermitian in a general case, the evolutionary operator,

(5) $$e^{-i\frac{t}{\hbar }H},$$

is not unitary. Therefore, the anti-PPH two-level system cannot be simulated directly by a normal method for a Hermitian system using one qubit only. Duality quantum algorithm provides a method to simulate the anti-PPH non-Hermitian system.

## 3. Duality Quantum Algorithm

Duality quantum algorithm was proposed in 2002 [71] for the first time, and it developed fast [72,73,74,75]. Because both the products of unitary operations and linear combinations of unitary (LCU) operations can be realized by duality quantum algorithm, it has a lot of applications to investigate novel quantum systems [16,17,18,19,20,21,76,77,78,79,80], e.g., systems of large-scale silicon quantum photonics, efficient quantum simulations of open quantum systems, quantum secure computing, passive quantum error correction, etc. Duality quantum algorithm has become one of the strongest tool in designing quantum algorithms [81]. Recently, scientists apply the method to design a full quantum algorithm for quantum chemistry simulation [82].

In the following sections, we show several optimized proposals, which are based on duality quantum algorithm, to simulate the anti-*P*-pseudo-Hermitian two-level system in different phases.

## 4. In an Eight-Dimensional Hilbert Space

First, we construct the anti-PPH two-level quantum system in an eight-dimensional Hilbert space, which can be realized by both a qubit-qudit hybrid system and a three-qubit system. We propose for both of the two systems. On one hand, it is more clear to demonstrate how to simulate the anti-PPH two-level system using the method of duality quantum algorithm by a qubit-qudit hybrid system. On the other hand, it is able to be implemented in a near-term qubit quantum computer.

### 4.1. Using a Qubit-Qudit Hybrid System

A work qubit *e* and an ancillary four-dimensional qudit *a* compose the hybrid system, extending an eight-dimensional Hilbert space. Similar to a qubit, a qudit can be used as a basic building block of a high-dimenisonal quantum computer. A superposition state of a four-dimensional qudit is a linear combination of four orthogonal logical bases |0〉, |1〉, |2〉 and |3〉, which can be realized by a four-level quantum system, e.g., four non-degenerate-energy levels of an ultra-cold atom, four split-levels of a nuclear spin, a particle with spin-$\frac{3}{2}$, etc. In some cases, qudits take advantages over qubits. For example, when solving the eigenvalue problem using quantum phase estimation algorithm, it can reach a higher accuracy by using qudits [83,84] than by using qubits [85].

**Figure 2:**
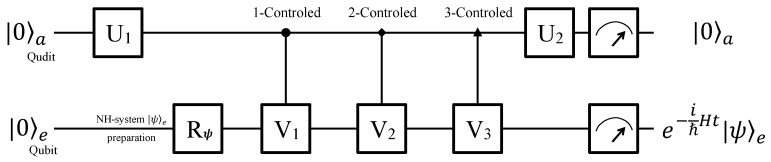
Quantum circuit for a qubit-qudit hybrid computer. The qubit-qudit hybrid system consists of an ancillary qudit and a work qubit, which will pass the quantum circuit from the left to the right. The system is initialized in ${|0\rangle }_{a}{|0\rangle }_{e}$ at first, and then the work qubit is prepared in arbitrary state ${|\psi \rangle }_{e}$ as demand. After being operated by a single-qudit rotation $U_{1}$, three controlled operations, i.e., 1-controlled $V_{1}$, 2-controlled $V_{2}$ and 3-controlled $V_{3}$, and a single-qudit rotation $U_{2}$, the the work qubit *e* will evolve as Equation (5) if the ancillary qudit is measured in state ${|0\rangle }_{a}$.

It is convenient to illustrate how to construct the anti-PPH system by using a qubit-qudit hybrid system, and the quantum circuit is shown in Figure 2. The work qubit will evolve as Equation (5) assisted by the ancillary qudit. At the beginning, the whole system is initialized to a pure state ${|0\rangle }_{a}{|0\rangle }_{e}$. A single rotation $U_{1}=\left[u_{jk}\right]$ in SO(4) (where k,j=1,2,3,4) is applied to the ancillary qudit, of which the explicit form is not unique as long as ${U_{1}}^{†}U_{1}=I_{4}$ and the elements in the first column satisfy $u_{k1}=\frac{a_{k}}{a_{0}}$ for k=1,2,3,4, where

(6) $$a_{1}=\frac{\left(e^{i\frac{\omega t}{2\hbar }}+e^{-i\frac{\omega t}{2\hbar }}\right)}{2}e^{\frac{t}{\hbar }r\cos\theta },$$

(7) $$a_{2}=-i\frac{\left(u+s\right)}{2\omega }\left(e^{i\frac{\omega t}{2\hbar }}-e^{-i\frac{\omega t}{2\hbar }}\right)e^{\frac{t}{\hbar }r\cos\theta },$$

(8) $$a_{3}=\frac{i(u-s)}{2\omega }\left(e^{i\frac{\omega t}{2\hbar }}-e^{-i\frac{\omega t}{2\hbar }}\right)e^{\frac{t}{\hbar }r\cos\theta },$$

(9) $$a_{4}=\frac{-ir\sin\theta }{\omega }\left(e^{i\frac{\omega t}{2\hbar }}-e^{-i\frac{\omega t}{2\hbar }}\right)e^{\frac{t}{\hbar }r\cos\theta },$$

and

(10) $$a_{0}=\sqrt{\sum_{k=1}^{4}a_{k}^{2}}=e^{\frac{t}{\hbar }r\cos\theta }\sqrt{1-\frac{{\left(u+s\right)}^{2}}{2\omega^{2}}{\left(e^{i\frac{\omega t}{2\hbar }}-e^{-i\frac{\omega t}{2\hbar }}\right)}^{2}}.$$

At the same time, a single qubit rotation $R_{\psi }$∈ SU(2) is applied to the work qubit to generate an arbitrary initial state ${|\psi \rangle }_{e}$ as demand, and ${|\psi \rangle }_{e}$ will evolve as the law charged by the anti-*P*-pseudo-Hermitian *H* in Equation (2). Three *k*-controlled gates follow, where k=1,2,3 indicates the logic state of the qudit, to construct the anti-PPH subsystem. The explicit forms of $V_{k}$’s (k=1,2,3) are

(11) $$V_{1}=\sigma_{x},$$

(12) $$V_{2}=i\sigma_{y},$$

and

(13) $$V_{3}=i\sigma_{z}.$$

Then, a single qudit rotation $U_{2}$, which is a Hadamard operator in SO(4), is applied to the ancillary qudit.

Now, the whole system evolves to a superposition state

(14) $$\frac{1}{2a_{0}}\left[{|0\rangle }_{a}\left(e^{-i\frac{t}{\hbar }H}\right){|\psi \rangle }_{e}+a_{0}\sum_{k=1}^{3}{|k\rangle }_{a}{|s_{k}\rangle }_{e}\right],$$

where the explicit forms of the states $|s_{k}\rangle_{e}$ (k=1,2,3) are not given because they are meaningless to the anti-PPH system. Finally, measurements are performed on the ancillary qubit. If the ancillary qudit is measured in one of the states ${|1\rangle }_{a}$, ${|2\rangle }_{a}$ or ${|3\rangle }_{a}$, we discard the results of the work qubit. In these cases, the system will be initialized to the pure state ${|0\rangle }_{a}{|0\rangle }_{e}$ again and the whole process above will be restarted. If the qudit is observed in state ${|0\rangle }_{e}$, the work qubit does evolve as the evolutionary operator charged by *H* in Equation (2) to a state of $e^{-i\frac{t}{\hbar }H}{|\psi \rangle }_{e}$. Noticing that the evolution operator in Equation (5) is not unitary, a normalizing factor is neglected here. Therefore, quantum simulation of the evolution of the anti-*P*-pseudo-Hermitian system is achieved in an indeterministic sense with a successful probability of

(15) $$\frac{1}{4a_{0}^{2}}\left[{}_{e}\langle \psi |\left(e^{i\frac{t}{\hbar }H^{†}}\right)\left(e^{-i\frac{t}{\hbar }H}\right){|\psi \rangle }_{e}\right].$$

### 4.2. Using Three Qubits

**Figure 3:**
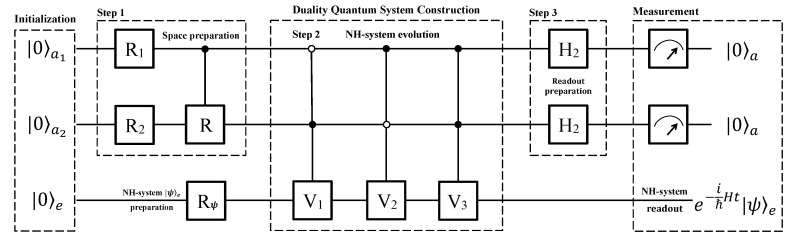
Quantum circuit designed for a quantum computer of three qubits using the full Hilbert space. Three qubits are essential for an arbitrary phase. Two of them are ancillary qubits and the third one is a work qubit. We divide the circuit into three blocks: (1) Initialization: the three qubits are initialized to ${|00\rangle }_{a}{|0\rangle }_{e}$, and then the work qubit is rotated to an arbitrary state ${|\psi \rangle }_{e}$ as demand. (2) Duality quantum system construction is composed of three steps. In the first step, two single-qubit rotations $R_{1}$, $R_{2}$ and a controlled operation *R* are applied to the two ancillary qubits, of which the effect is similar to $U_{1}$ in Figure 2. In the second and third steps, three controlled-controlled gates and two Hadamard gates are used. (3) Measurement: the work qubit will evolve as the law charged by the anti-PPH Hamiltonian *H* if the ancillary qubits output ${|00\rangle }_{a}$.

**Figure 4:**
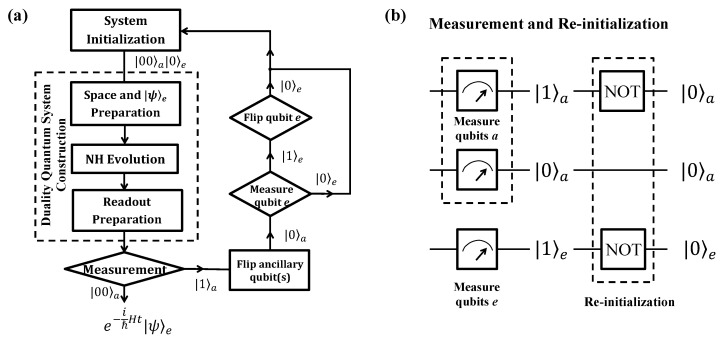
Flowchart and re-initialization. (**a**) Flowchart of quantum computers to simulate the anti-PPH system. The whole process is divided into three main blocks for clearness, which are system initialization, duality quantum system construction, and measurement. (**b**) Quantum circuit to re-initialize the system. If the two ancillary qubits are not measured in state ${|00\rangle }_{a}$, the whole system will be initialized to state ${|00\rangle }_{a}{|0\rangle }_{e}$ again as the circuit. A flip operation is applied to each qubit with |1〉-output, while no operation is applied to that with |0〉-output.

Now we show how to simulate the anti-PPH two-level system in a near-term qubit quantum computer that has become an available technology. For illustration, the quantum circuit is shown in Figure 3 and the flowchart of the quantum computer program is shown in Figure 4a, including three blocks of system initialization, construction and measurement.

In the process of system initialization, the whole system consisting of two ancillary qubits and one work qubit is initialized to a pure state ${|00\rangle }_{a}{|0\rangle }_{e}$, and then a single qubit rotation $R_{\psi }$ is applied to the work qubit to obtain an arbitrary state ${|\psi \rangle }_{e}$ as demand. The second block is divided into three steps, i.e., the space preparation, system evolution, and readout preparation.

First, two single-qubit rotation $R_{1}$ and $R_{2}$ are applied to the first and second ancillary qubits, respectively, where

(16) $$R_{1}=\frac{1}{a_{0}}\left(\begin{array}{cc} \sqrt{a_{1}^{2}+a_{2}^{2}} & -\sqrt{a_{3}^{2}+a_{4}^{2}}\\ \sqrt{a_{3}^{2}+a_{4}^{2}} & \sqrt{a_{1}^{2}+a_{2}^{2}} \end{array}\right)$$

and

(17) $$R_{2}=\frac{1}{\sqrt{a_{1}^{2}+a_{2}^{2}}}\left(\begin{array}{cc} a_{1} & -a_{2}\\ a_{2} & a_{1} \end{array}\right).$$

Then, a 1-controlled *R* is followed, where

(18) $$R={R_{2}}^{-1}R_{3},$$

and

(19) $$R_{3}=\frac{1}{\sqrt{a_{3}^{2}+a_{4}^{2}}}\left(\begin{array}{cc} a_{3} & -a_{4}\\ a_{4} & a_{3} \end{array}\right).$$

The above three operations prepare the space, which have a similar effect as $U_{1}$ in Figure 2.

Then, three jointly controlled $V_{k}$’s (k=1,2,3) gates, i.e., the two ancillary qubits control the work qubit jointly, and two Hadamard gates follow. The matrices $V_{k}$’s (k=1,2,3) are the same as that in Equations (11)–(13). The two Hadamard operators are applied to the two ancillary qubits, respectively.

Now, the whole system evolves to a superposition state

(20) $$\frac{1}{2a_{0}}\left[{|00\rangle }_{a}\left(e^{-i\frac{t}{\hbar }H}\right){|0\rangle }_{e}+a_{0}\sum_{kj=01}^{10,11}{|kj\rangle }_{a}{|s_{kj}\rangle }_{e}\right],$$

where we do not give the explicit forms of the states $|s_{kj}\rangle_{e}$ (kj=01,10,11) for the same reason that they are meaningless to the anti-PPH system and we will discard the results.

Finally, measurements are performed on the two ancillary qubits. If the two ancillary qubits is in state ${|00\rangle }_{a}$, the work qubit will evolve to $e^{-i\frac{t}{\hbar }H}{|0\rangle }_{e}$ as the evolutionary operator in Equation (5) of the anti-PPH system. If the two ancillary qubits are measured in one of the states ${|kj\rangle }_{a}$ (kj=01,10,11), the simulation will be terminated and the system will be re-initialized to ${|00\rangle }_{a}{|0\rangle }_{e}$ as the quantum circuit shown in Figure 4b. The process will be started over until the output ${|00\rangle }_{a}$ is obtained. Therefore, it is an indeterministic way to achieve the quantum simulation. The successful probability is equal to that in Equation (15).

## 5. In a Six-Dimensional Hilbert Space

The anti-PPH two-level quantum system can be simulated more efficiently in a smaller Hilbert space of six-dimensions with a higher successful probability. Similar to the eight-dimensional method, the six-dimensional Hilbert space can be realized by a qubit-qutrit hybrid system or by a subspace of three qubits.

### 5.1. Using a Qubit-Qutrit Hybrid System

The hybrid system is composed of a work qubit *e* and an ancillary qutrit *a*, by which a six-dimensional Hilbert space is extended. The ancillary qutrit assists the work qubit to evolve as Equation (5) in a probabilistic way. A qutrit has three independent logic state |0〉, |1〉 and |2〉, and any superposition state is a linear combination of them. As a building block of a higher-dimensional quantum computer, it can be realized by a three-level physical system, such as three energy levels of an ultra-cold atom, three split-levels of a nuclear spin, a particle with spin-1, etc.

**Figure 5:**
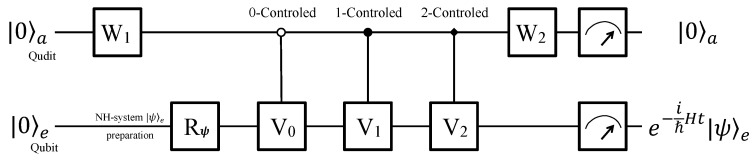
Quantum circuit for a qubit-qutrit hybrid computer. The hybrid system consists of an ancillary qutrit and a work qubit. The system is initialized to state ${|0\rangle }_{a}{|0\rangle }_{e}$ at first, and the work qubit is rotated to an arbitrary state ${|\psi \rangle }_{e}$ as demand. After being operated by a single-qutrit rotation $W_{1}$, three controlled operations and a single-qutrit rotation $W_{2}$, the the work qubit *e* will evolve as the law charged by the anti-PPH Hamiltonian if the ancillary qutrit outputs ${|0\rangle }_{a}$.

The quantum circuit is shown in Figure 5 to illustrate how to achieve the simulation. First of all, the whole system is prepared in a pure state ${|0\rangle }_{a}{|0\rangle }_{e}$, and $R_{\phi }$ rotates the work qubit to arbitrary ${|\psi \rangle }_{e}$ as needed. Meanwhile, $W_{1}\in$ SO(3) is applied to the ancillary qutrit

(21) $$W_{1}=\frac{1}{c}\left(\begin{array}{ccc} c_{1} & -\frac{cc_{3}}{\sqrt{c_{1}^{2}+c_{3}^{2}}} & \frac{c_{1}c_{2}}{\sqrt{c_{1}^{2}+c_{3}^{2}}}\\ c_{2} & 0 & -\sqrt{c_{1}^{2}+c_{3}^{2}}\\ c_{3} & \frac{cc_{1}}{\sqrt{c_{1}^{2}+c_{3}^{2}}} & \frac{c_{2}c_{3}}{\sqrt{c_{1}^{2}+c_{3}^{2}}} \end{array}\right),$$

where

(22) $$c_{1}=\sqrt{1-\frac{us}{\omega^{2}}{\left(e{}^{i\frac{\omega t}{2\hbar }}-e^{-i\frac{\omega t}{2\hbar }}\right)}^{2}}\cdot e^{\frac{t}{\hbar }r\cos\theta },$$

(23) $$c_{2}=-i\frac{\left(u+s\right)}{2\omega }\left(e^{i\frac{\omega t}{2\hbar }}-e^{-i\frac{\omega t}{2\hbar }}\right)e^{\frac{t}{\hbar }r\cos\theta },$$

(24) $$c_{3}=i\frac{\left(u-s\right)}{2\omega }\left(e^{i\frac{\omega t}{2\hbar }}-e^{-i\frac{\omega t}{2\hbar }}\right)e^{\frac{t}{\hbar }r\cos\theta },$$

and

(25) $$c=\sqrt{\;\sum_{k=1}^{3}c_{k}^{2}\;}=a_{0}.$$

It is not difficult to check that $c_{k}$’s (k=1,2,3) and *c* are real numbers no matter whether ω is real or imaginary. Then, three controlled operations, $C_{0-V_{0}}$, $C_{1-V_{1}}$ and $C_{2-V_{2}}$, follow. $C_{k-V_{k}}$’s are *k*-controlled gates, acting $V_{k}$ to the work qubit *e* when the ancillary qutrit is in state ${|k\rangle }_{a}$, where k=0, 1 and 2. The explicit forms of them are

(26) $$C_{0-V_{0}}=\left(\begin{array}{ccc} V_{0} & 0 & 0\\ 0 & I_{2} & 0\\ 0 & 0 & I_{2} \end{array}\right),$$

where

(27) $$V_{0}=\left(\begin{array}{cc} e^{i\phi } & 0\\ 0 & e^{-i\phi } \end{array}\right),$$

is an element of SU(2), in which the angle ϕ is decided by

(28) $$\cos\phi =\frac{e{}^{i\frac{\omega t}{2\hbar }}+e^{-i\frac{\omega t}{2\hbar }}}{2\sqrt{1-\frac{us}{\omega^{2}}{\left(e{}^{i\frac{\omega t}{2\hbar }}-e^{-i\frac{\omega t}{2\hbar }}\right)}^{2}}}$$

and

(29) $$\sin\phi =\frac{-i(e{}^{i\frac{\omega t}{2\hbar }}-e^{-i\frac{\omega t}{2\hbar }})r\sin\theta }{\omega \sqrt{1-\frac{us}{\omega^{2}}{\left(e{}^{i\frac{\omega t}{2\hbar }}-e^{-i\frac{\omega t}{2\hbar }}\right)}^{2}}};$$

(30) $$C_{1-V_{1}}=\left(\begin{array}{ccc} I_{2} & 0 & 0\\ 0 & V_{1} & 0\\ 0 & 0 & I_{2} \end{array}\right),$$

and

(31) $$C_{2-V_{2}}=\left(\begin{array}{ccc} I_{2} & 0 & 0\\ 0 & I_{2} & 0\\ 0 & 0 & V_{2} \end{array}\right),$$

where $V_{1}=\sigma_{x}$ and $V_{2}=i\sigma_{y}$ in accordance with Equations (11) and (12); $I_{2}$ is the unit element of SU(2).

Finally, a single qutrit operation

(32) $$W_{2}=\frac{1}{\sqrt{3}}\left(\begin{array}{ccc} 1 & \sqrt{\frac{3}{2}} & \frac{1}{\sqrt{2}}\\ 1 & 0 & -\sqrt{2}\\ 1 & -\sqrt{\frac{3}{2}} & \frac{1}{\sqrt{2}} \end{array}\right)$$

is applied to the ancillary qutrit *a*. After operated by the series of operations above, the input state ${|0\rangle }_{a}{|\psi \rangle }_{e}$ evolves to

(33) $$\frac{1}{\sqrt{3}c}\left[{|0\rangle }_{a}\left(e^{-i\frac{t}{\hbar }H}\right){|\psi \rangle }_{e}+c\left({|1\rangle }_{a}|s_{1}\rangle_{e}+{|2\rangle }_{a}{|s_{2}\rangle }_{e}\right)\right],$$

where the normalized states $|s_{1}\rangle$ and $|s_{2}\rangle$ are not shown explicitly because the results of work qubit will be discarded on the condition that the ancillary qutrit is in state ${|1\rangle }_{a}$ or ${|1\rangle }_{a}$.

The qubit-qutrit hybrid system is measured now. If the ancillary qutrit *a* is observed in state ${|0\rangle }_{a}$, the work qubit *e* will evolve to a state $e^{-i\frac{t}{\hbar }H}{|\psi \rangle }_{e}$ that entangled with ${|0\rangle }_{a}$. In this case, the evolutionary law of the work qubit is charged by the anti-PPH *H* in Equation (2) as we want. Given that the *H* in Equation (2) is non-Hermitian, the quantum simulation is in a probabilistic way only when the ancillary qutrit outputs state ${|0\rangle }_{a}$. In the other two cases when the ancillary qutrit collapses into state ${|1\rangle }_{a}$ or ${|2\rangle }_{a}$, the work qubit subsystem will not evolve as Equation (5) and we will discard the results of the work qubit. If the ancillary qutrit fails to output ${|0\rangle }_{a}$, the process will be terminated and reset to the beginning. Then the quantum simulation is restarted until ${|0\rangle }_{a}$ is observed. Although it is not a deterministic method, it works to simulate the non-unitary evolution of the anti-PPH two-level quantum system.

We now analyze the parameter $c=a_{0}$ in Equation (25) and calculate the successful probability. It is not difficult to check that *c* is always a real number and not less than 1, and this is reasonable noticing that the evolution operator $e^{-i\frac{t}{\hbar }H}$ in Equation (5) is not unitary. In fact, *c* renormalizes the whole system unitary because of the Hermiticity of the qubit-qutrit hybrid system. When the dynamic parameters of *H* satisfy that r·cosθ and s+u=0, *H* will become Hermitian, which means that a phase transition happens in the parametric space. In this case, the parameter *c* is equal to 1 and the time evolutionary operator $e^{-i\frac{t}{\hbar }H}$ becomes unitary. It can be seen clearly from the final state in Equation (33) that becomes $\frac{1}{\sqrt{3}}\left[{|0\rangle }_{a}\left(e^{-i\frac{t}{\hbar }H}\right){|\psi \rangle }_{e}+{|1\rangle }_{a}|s_{1}\rangle_{e}+{|2\rangle }_{a}{|s_{2}\rangle }_{e}\right]$. According to Equations (25) and (33), the successful possibility to measure the ancillary qutrit in state ${|0\rangle }_{a}$ is

(34) $$\frac{1}{3a_{0}^{2}}\left[{}_{e}\langle \psi |\left(e^{i\frac{t}{\hbar }H^{†}}\right)\left(e^{-i\frac{t}{\hbar }H}\right){|\psi \rangle }_{e}\right],$$

depending on both the *H* in Equation (2) and the initial state of the work qubit together.

Compared to Equation (15), the value of Equation (34) is higher. Therefore, the successful probability of the method here using six dimensions is higher than that using eight dimensions. Furthermore, the successful probability here is also higher than that of the previous work using more measurements [20], which is equal to Equation (34) times a parameter-dependent variable less than one.

### 5.2. Using Three Qubits

**Figure 6:**
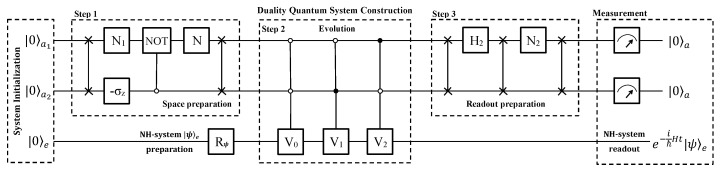
Quantum circuit designed for a quantum computer of three qubits using six dimensional subspace. Two qubits are ancillary and the third one is a work qubit. The process can be divided into three blocks: system initialization, duality quantum system construction, and measurement. The second block includes three steps of space preparation, evolution and readout preparation. After measurement, the work qubit will evolve as the anti-PPH Hamiltonian if the ancillary subsystem is read out as ${|00\rangle }_{a}$. Refer to the text for details of the operations.

To run in a near-term quantum computer, it is necessary to show how to achieve the efficient simulation using qubits only. The whole process is illustrated by the flowchart show in Figure 4a, and the quantum circuit is shown in Figure 6. Although three qubits are essential to this method yet, it uses a six-dimensional subspace of the whole Hilbert space. The successful probability is higher than that using the full space of the three qubits.

In detail, the first two qubits take a similar role as that of the ancillary qutrit in the previous section, while the third one takes the same role as the work qubit. A six-dimensional subspace of the three qubits are used during the process, which is spanned by six logic bases ${|00\rangle }_{a}{|0\rangle }_{e}$, ${|01\rangle }_{a}{|0\rangle }_{e}$, ${|10\rangle }_{a}{|0\rangle }_{e}$, ${|00\rangle }_{a}{|1\rangle }_{e}$, ${|01\rangle }_{a}{|1\rangle }_{e}$, and ${|10\rangle }_{a}{|1\rangle }_{e}$.

We illustrate the proposal by the quantum circuit shown in Figure 6 now. In the first block, the three qubits are initialized to a pure state ${|00\rangle }_{a}{|0\rangle }_{e}$, and the work qubit *e* is prepared to arbitrary state ${|\psi \rangle }_{e}$ by $R_{\psi }$ as needed.

In the first step of the block to construct the duality quantum system, the six-dimensional subspace spanned by bases ${|jk\rangle }_{a}{|m\rangle }_{e}$ (jk=00,01,10, m=0,1) is prepared. The two ancillary qubits are swapped at first. Two single qubit gates $N_{1}$ and $-\sigma_{z}$ are applied to the first and second ancillary qubits, respectively. Next, we perform a 0-controlled NOT gate, which the first and second ancillary qubits are the target and control qubits, respectively. Next, a single qubit *N* rotates the first ancillary qubit. The explicit form of $N_{1}$ and *N* are

(35) $$N_{1}=\frac{1}{c}\left(\begin{array}{cc} c_{2} & -\sqrt{c_{1}^{2}+c_{3}^{2}}\\ \sqrt{c_{1}^{2}+c_{3}^{2}} & c_{2} \end{array}\right),$$

and

(36) $$N=\frac{1}{\sqrt{c_{1}^{2}+c_{3}^{2}}}\left(\begin{array}{cc} c_{3} & c_{1}\\ -c_{1} & c_{3} \end{array}\right),$$

where the parameters $c_{k}$’s (k= 1, 2, 3) are that in Equations (22)–(24). At last of this step, the two ancillary qubits are swapped again. These operations achieve the space preparation, guaranteeing that no ${|11\rangle }_{a}$ is involved. The total effect of the operations in this step is similar to that of $W_{1}$ in Figure 5.

In the second step of this block, the work qubit is controlled by the two ancillary qubits jointly. In detail, three jointly controlled gates are used as shown in Figure 6, and they are the 00-controlled $V_{0}$ gate, 01-controlled $V_{1}$ and 10-controlled $V_{2}$. The explicit form of $V_{k}$’s (k=0,1,2) are the same as Equation (27), Equation (11) and Equation (12), respectively.

In the third step, three SWAP gates are performed on the two ancillary qubits, and they are separated by two single-qubit operations applied to the first ancillary qubit, which are a Hadamard $H_{2}$ and an

(37) $$N_{2}=\frac{1}{\sqrt{3}}\left(\begin{array}{cc} \sqrt{2} & 1\\ 1 & -\sqrt{2} \end{array}\right).$$

Now the whole system evolves to a superposition state

(38) $$\frac{1}{\sqrt{3}c}\left[{|00\rangle }_{a}\left(e^{-i\frac{t}{\hbar }H}\right){|\psi \rangle }_{e}+c\sum_{kj=01,10}{|kj\rangle }_{a}{|s_{kj}\rangle }_{e}\right],$$

noticing that ${|11\rangle }_{a}$ is not involved in the whole process.

Finally, measurements are performed on the ancillary qubits. If ${|00\rangle }_{a}$ of the ancillary subsystem is observed, the work qubit will evolve as Equation (5) to a state $e^{-i\frac{t}{\hbar }H}{|\psi \rangle }_{e}$. If either of the two ancillary qubits outputs ${|1\rangle }_{a}$, i.e., ${|01\rangle }_{a}$ or ${|10\rangle }_{a}$, the work qubit will evolve to some states $|s_{kj}\rangle_{e}$ (kj= 01, 10). In these cases, we discard the results of the work qubit, and the simulating process is terminated to restart as the quantum circuit shown in Figure 4b. The successful probability is equal to Equation (34), which is higher than that of the method using the whole space of the 3-qubit system and the previous work [20].

## 6. In a Four- and a Two-Dimensional Hilbert Spaces

In this section, we investigate special phases of the anti-PPH Hamiltonian, including an anti-PT-symmetric and a Hermitian phases. In the special phases, the anti-PPH two-level system can be simulated in smaller Hilbert spaces by fewer qubits. It is helpful to improve the efficiency and reduce the difficulty of experimental operations.

### 6.1. Using Two Qubits

**Figure 7:**
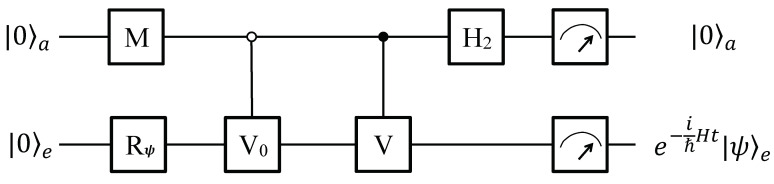
Quantum circuit for special phases using two qubits. The system consists of an ancillary and a work qubits. It is initialized to state ${|0\rangle }_{a}{|0\rangle }_{e}$ at the beginning, and the work qubit is prepared to an arbitrary state ${|\psi \rangle }_{e}$ by a single qubit rotation $R_{\psi }$ as needed. Then, a single-qubit rotation *M*, two controlled operations (0-controlled $V_{0}$ and 1-controlled *V*), and a Hadamard follow. If the ancillary qubit is observed as ${|0\rangle }_{a}$, the work qubit *e* will evolve as Equation (5) when *H* is in some special phases (s±u=0), including an anti-PT-symmetric phase (-). *M* and *V* have different explicit forms in the two cases (±).

When the dynamic parameters satisfy s+u=0 or s−u=0, $c_{2}$ or $c_{3}$ becomes zero, respectively. In these cases, it is able to simulate the anti-*P*-pseudo-Hermitian two-level system in a four-dimensional Hilbert space using only two qubits by our method. At the beginning, the system is initialized to a pure state ${|0\rangle }_{a}{|0\rangle }_{e}$, and the work qubit is rotate to state ${|\psi \rangle }_{e}$ as needed. Then, a single-qubit rotations *M*, a 0-controlled $V_{0}$, an 1-controlled *V*, and a Hadamard $H_{2}$ in SU(2) are applied in order as the quantum circuit shown in Figure 7, where $V_{0}$ has the same form of that in Equation (27).

(i) In the case of s+u=0, the explicit form of matrices *M* and *V* in Figure 7 are

(39) $$\frac{1}{\sqrt{{c_{1}}^{2}+{c_{3}}^{2}}}\left(\begin{array}{cc} c_{1} & -c_{3}\\ c_{3} & c_{1} \end{array}\right),$$

and $i\sigma_{y}$, respectively.

(ii) In the case of s−u=0, *H* in Equation (2) satisfies PTH+HPT=0, which is the relation of anti-PT symmetry. This means the anti-PPH Hamiltonian in Equation (2) is in its anti-PT-symmetric phase now. The explicit form of matrices *M* and *V* are

(40) $$\frac{1}{\sqrt{{c_{1}}^{2}+{c_{3}}^{2}}}\left(\begin{array}{cc} c_{1} & -c_{3}\\ c_{3} & c_{1} \end{array}\right),$$

and $\sigma_{x}$, respectively.

For both of the two cases, the parameters $c_{k}$’s (k= 1, 2, 3) have been shown in Equations (22)–(24). Now, the initial state will evolve to a final superposition state

(41) $$\frac{1}{\sqrt{2}c}\left[{|0\rangle }_{a}\left(e^{-i\frac{t}{\hbar }H}\right){|\psi \rangle }_{e}+{c|1\rangle }_{a}{|s_{1}^{\prime }\rangle }_{e}\right],$$

where $c=\sqrt{c_{1}^{2}+c_{3}^{2}}$ or $\sqrt{c_{1}^{2}+c_{2}^{2}}$ for the two cases of (i) s+u=0 or (ii) s−u=0, which is accordant with Equations (23)–(25). When measurements are performed, the work qubit *e* will evolve to $e^{-i\frac{t}{\hbar }H}{|0\rangle }_{e}$ if the ancillary qubit outputs ${|0\rangle }_{a}$ with a probability of

(42) $$\frac{1}{2c^{2}}\left[{}_{e}\langle \psi |\left(e^{i\frac{t}{\hbar }H^{†}}\right)\left(e^{-i\frac{t}{\hbar }H}\right){|\psi \rangle }_{e}\right].$$

If the ancillary qubit is observed in state ${|1\rangle }_{a}$, the work qubit will evolve to some state $|s_{1}^{\prime }\rangle_{e}$ and we will discard the result. Therefore, the previous work [19] can be achieved more efficiently by fewer qubits with higher successful probability.

### 6.2. Using One Qubit

When r·cosθ=0 and s+u=0, *H* becomes $\left(\begin{array}{cc} -r\sin\theta & is\\ -is & r\sin\theta \end{array}\right)$ and it is Hermitian. In this Hermitian phase, the parameter *c* is equal to 1 and the time evolutionary operator $e^{-i\frac{t}{\hbar }H}$ becomes unitary. Therefore, the system in this phase can be simulated using only one qubit in a deterministic way.

## 7. Discussion for Experimental Implementation

To implement in a near-term quantum computer, three qubits are enough to simulate the anti-*P* pseudo-Hermitian two-level system in arbitrary phase. Both the six- and the eight-dimension proposals are able to realize the quantum simulation. It depends on the stability and controllability of the experimental systems to adopt which one of the two proposals.The six-dimension proposal has a larger successful probability but uses more two-qubit gates in the parts of space and measurement preparation than the eight-dimension one. Therefore, the six-dimension proposal is good for aiming at a higher successful probability, while the eight-dimension proposal is appropriate to an experimental system of which the accumulated errors induced by two-qubit gates are big. Fewer qubits are able to simulate the anti-PPH system in the anti-PT-symmetric phase, Hermitian phase, etc., using the four- or two-dimension proposals.

For experimental realization, we take a nuclear-magnetic-resonance (NMR) system as an example. Three nuclei of spin-$\frac{1}{2}$ take the role of the work and ancillary qubits. The spatial-averaging method can be used [86] to prepare the initial pseudo-pure state ${|00\rangle }_{a}{|0\rangle }_{e}$, and sequences of magnetic pulses are designed to realize the the quantum gates. For example, a single qubit rotation can be realized by a series of hard pulses, while two-qubit operations can be achieved by combinations of hard pulses and free evolutions of two nuclei of spin-$\frac{1}{2}$ in periods of time [12]. In the anti-PT-symmetric phase, two spins are used as the work qubit and the ancillary qubit. In the Hermitian phase, only one spin qubit is needed for the quantum simulation. In this case, linear quantum optics system is also a candidate for the experimental simulation. The two orthogonal polarized directions of a photon are state |0〉 and |1〉. A single qubit operation is realized by a series of half-wave and quarter-wave plates [87]. Although it is possible in principle to realize a two-polarization-qubit operation using measurement induced nonlinearity [88,89], the efficiency is low especially when there are a lot of two-qubit gates.

## 8. Conclusions

We investigate quantum simulations of a time-independent anti-*P*-pseudo-Hermitian two-level system in different phases. The time evolutions of the system in different phases from arbitrary initial state can be simulated efficiently by related proposals based on the method of duality quantum computing. To implement in a qubit computer, three qubits are essential to achieve the quantum simulation in an arbitrary phase. Depending on the output of the two ancillary qubits, the work qubit evolves as the law of the anti-PPH system in an indeterministic way. Both six- and eight-dimension proposals are able to achieve the simulation and have different advantages. The six-dimension proposal has a higher successful probability, while the eight-dimension proposal needs fewer two-qubit operations. Therefore, it depends on the stability and controllability of an experimental system to choose which proposal. The anti-PPH system can be simulated by fewer qubits when it is in some special phases. For example, the system can be simulated by two qubits in the anti-PT-symmetric phase, and one qubit is enough in the Hermitian phase. We recommend to experimentally implement in NMR when the anti-PPH system is in an arbitrary phase or in the anti-PT-symmetric phase. In the Hermitian phase, both NMR and quantum optics are efficient to simulate the system. The flowchart and quantum circuits are given for experimental implementations in a near-term quantum computer, and they can be optimized in practice. Future experiments and phenomena related to the anti-PPH two-level system in different phases can be designed and investigated in quantum level based on our proposals.

## Abbreviations

The following abbreviations are used in this manuscript:

anti-PPH	anti-*P*-pseudo-Hermitian
NH	non-Hermitian
EP	exceptional point
NMR	nuclear magnetic resonance
